# Combined Toxicity of Gas Plasma Treatment and Nanoparticles Exposure in Melanoma Cells In Vitro

**DOI:** 10.3390/nano11030806

**Published:** 2021-03-22

**Authors:** Sander Bekeschus

**Affiliations:** ZIK *plasmatis*, Leibniz Institute for Plasma Science and Technology (INP), Felix-Hausdorff-Str. 2, 17489 Greifswald, Germany; sander.bekeschus@inp-greifswald.de; Tel.: +49-3834-554-3948

**Keywords:** B16, gas plasma technology, kINPen, plasma medicine, reactive oxygen and nitrogen species, RNS, ROS, SK-MEL-28

## Abstract

Despite continuous advances in therapy, cancer remains a deadly disease. Over the past years, gas plasma technology emerged as a novel tool to target tumors, especially skin. Another promising anticancer approach are nanoparticles. Since combination therapies are becoming increasingly relevant in oncology, both gas plasma treatment and nanoparticle exposure were combined. A series of nanoparticles were investigated in parallel, namely, silica, silver, iron oxide, cerium oxide, titanium oxide, and iron-doped titanium oxide. For gas plasma treatment, the atmospheric pressure argon plasma jet kINPen was utilized. Using three melanoma cell lines, the two murine non-metastatic B16F0 and metastatic B16F10 cells and the human metastatic B-Raf mutant cell line SK-MEL-28, the combined cytotoxicity of both approaches was identified. The combined cytotoxicity of gas plasma treatment and nanoparticle exposure was consistent across all three cell lines for silica, silver, iron oxide, and cerium oxide. In contrast, for titanium oxide and iron-doped titanium oxide, significantly combined cytotoxicity was only observed in B16F10 cells.

## 1. Introduction

Despite continuous advances in therapy, cancer remains a deadly condition. In 2020 in the US alone, 1.8 Mio new cancer cases were diagnosed, while more than 600,000 people died due to the malignant disease [1]. Among all types of cancers, skin cancer has the highest incidence globally, especially in Caucasians [2]. Typical risk factors include solar radiation, age, and skin phototype [3]. The highest mortality among skin cancer is attributed to malignant metastatic melanoma. This type of cancer has the highest mutation rate among all malignancies [4], making it especially difficult to treat because therapy-resistance often arises against many types of therapeutic approaches. This is also why melanoma is often used as a so-called model tumor, meaning that if therapies succeed against melanoma, they are likely to perform well in other cancer entities, too. The onset of the new age and checkpoint-immunotherapy success is an excellent example of this [5], and the state-of-the-art views on melanoma diagnosis and therapy have been outlined recently [6,7,8]. Nevertheless, many patients do not benefit from therapy, establishing the need to optimize treatment strategies further.

In the past years, gas plasma technology emerged as a novel tool to target tumors, especially skin [9]. It mainly acts via the deposition of reactive oxygen and nitrogen species (ROS/RNS), placing the field of plasma medicine—which has been reviewed recently [10,11,12]—in the heart of redox biology [13]. Several studies have underlined the anti-melanoma effects of gas plasma treatment in vitro and in vivo [14,15,16]. A more established innovative anti-melanoma approach is the use of nanotherapeutics such as nanoparticles [17]. The benefit of nanoparticles is the broad types of materials used together with a range of different functionalizations [18]. Their putative application for medical purposes has been recently summarized [19,20,21]. We here combined gas plasma treatment with nanoparticle exposure in three melanoma cell lines and found combined cytotoxicity in five out of seven nanoparticle types investigated.

## 2. Materials and Methods

### 2.1. Cell Culture

The melanoma cell lines B16F0 (ATCC CRL-6322, non-metastatic cell line), B16F10 (ATCC CRL-6475, metastatic cell line), and SK-MEL-28 (ATCC HTB-72, metastatic cell line) were cultured in Roswell Park Memorial Institute (RPMI1640) medium supplemented with 10% fetal bovine serum, 2% penicillin/streptomycin, and 1% glutamine (all Sigma, Taufkirchen, Germany). Cell lines were split two to three times per week and incubated under standard conditions at 37 °C, 95% humidity, and 5% CO_2_ (Binder, Tuttlingen, Germany).

### 2.2. Nanoparticle and Plasma Treatment

Nanoparticle acquisition or preparation and solvation was done as previously reported [22]. One day before the experiment, 1 × 10^4^ cells were seeded in 100 µL of fully supplemented cell culture medium in 96-well flat-bottom culture plates (Nunc, Roskilde, Denmark) and allowed to adhere overnight. Before treatment, the cell culture medium was taken off, and 50 µL of fresh and fully supplemented cell culture medium was added. The nanoparticles used in this study (Table 1) were silica nanoparticles, both small (Si_30_) and larger sizes (Si_150_), and metal oxide nanoparticles, including silver (Ag), iron oxide (FeO), cerium oxide (CeO_2_), titanium oxide (TiO_2_), and 10% iron-doped titanium oxide (FeTiO_2_). All nanoparticles were stored at 4 °C and thoroughly vortexed before their use. Nanoparticles were prepared at indicated concentrations in 50 µL of fully supplemented cell culture medium and added to the cells at the final concentration indicated in the figure legend and immediately before plasma treatment. For plasma treatment, the atmospheric pressure argon plasma jet kINPen was used with argon (99.9999% pure; Air Liquide, Greifswald, Germany) as feed gas at two standard liters per minute. The jet was operated at a frequency of 1 MHz, and its technical details have been reviewed before [23,24]. For the cells’ treatment, the jet was attached to a computer-controlled *xyz* motorized stage with predetermined parameters for the center position, treatment height, and treatment times for each of the wells as recently described [25].

### 2.3. Microscopy

Selected conditions were imaged using an Operetta CLS high-content imaging device (PerkinElmer, Hamburg, Germany). Brightfield and digital phase contrast were imaged using a 5x air objective (NA: 0.16; Zeiss, Jena, Germany) and overlaid for display.

### 2.4. Metabolic Activity

To analyze metabolic activity, 50 µL of fully supplemented cell culture medium containing resazurin (Sigma, Taufkirchen, Germany) at a final concentration of 100 µM was added to the cells. Resazurin freely diffuses into cells, where it is reduced by nicotinamide adenine dinucleotide phosphate (NADPH) to its fluorescent product, resorufin. After 4 h of incubation, the plate was transferred to an F200 microplate reader (Tecan, Männdedorf, Switzerland), and resorufin fluorescence was quantified at λ_ex_ 535 nm and λ_em_ 590 nm. For each cell line, the relative fluorescence units obtained were normalized to the respective untreated controls.

### 2.5. ROS Quantification

For analysis of the short-lived ROS deterioration product hydrogen peroxide (H_2_O_2_), 100 µL of phosphate-buffered saline (PBS) was exposed to gas plasma as described above. To control the assay’s specificity, N-acetylcysteine (NAC, 2 mM; Sigma, Taufkrichen, Germany) or catalase (5 µg/mL; Sigma, Taufkirchen, Germany) was added before plasma treatment to scavenge ROS and H_2_O_2_, respectively. Untreated and argon (Ar) gas-treated PBS was used as negative controls. Quantification with amplex ultra red (ThermoFisher, Bremen, Germany) was done as described before [26].

## 3. Results

**Figure 1:**
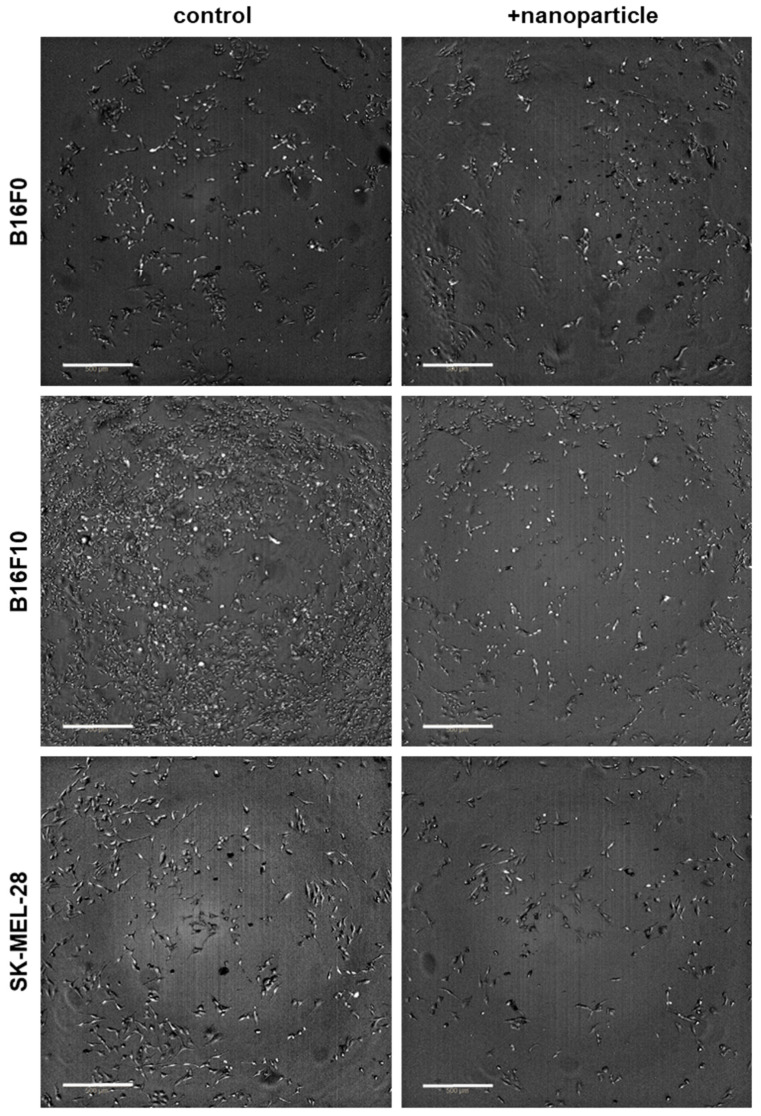
Microscopic images of melanoma cell lines used in this study. Shown are images of B16F0, B16F10, and SK-MEL-28 cells exposed to TiO_2_ particles. The cells were untreated (**left** images) or exposed to nanoparticles (**right** images). Images were acquired 16 h later and show overlays of brightfield and digital phase-contrast imaging. Scale bar = 500 µm.

**Figure 2:**
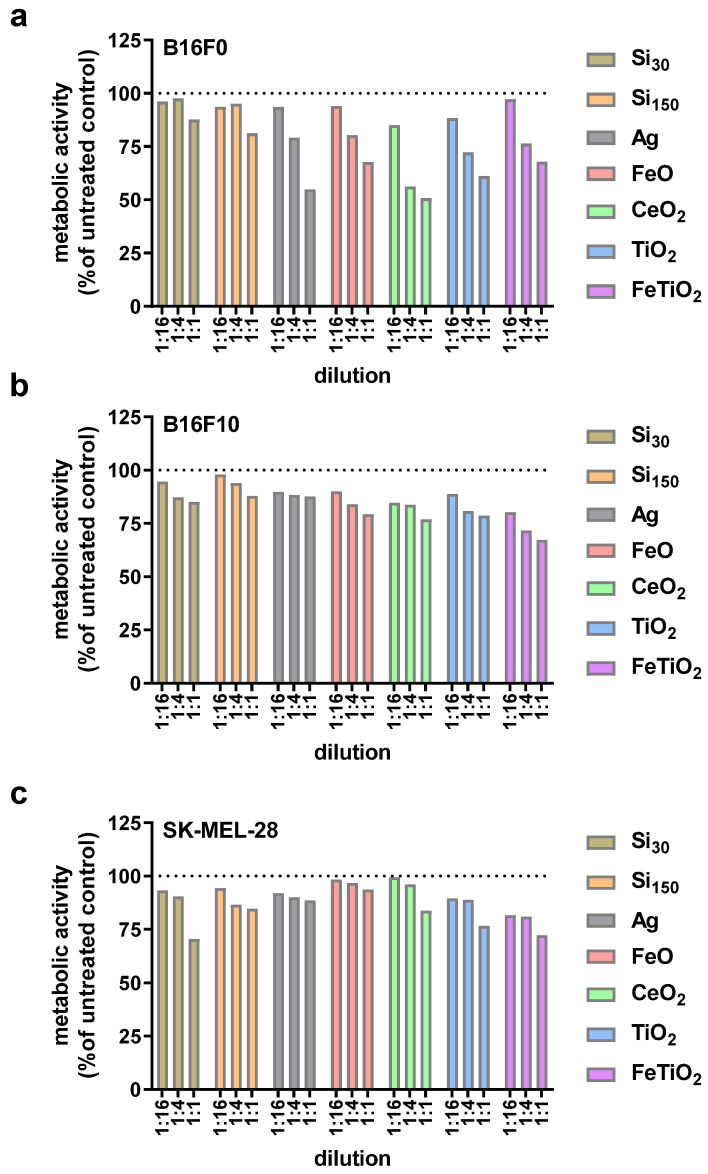
Concentration-dependent effects of nanoparticles. B16F0 (**a**), B16F10 (**b**), and SK-MEL-28 (**c**) cells were cultured with nanoparticles or were left untreated. At 24 h, the metabolic activity was determined using a resazurin-based assay, and data were normalized to that of untreated control cells. The top particle concentration (1:1) was 10 µg/mL. Data are from one pilot experiment.

**Figure 3:**
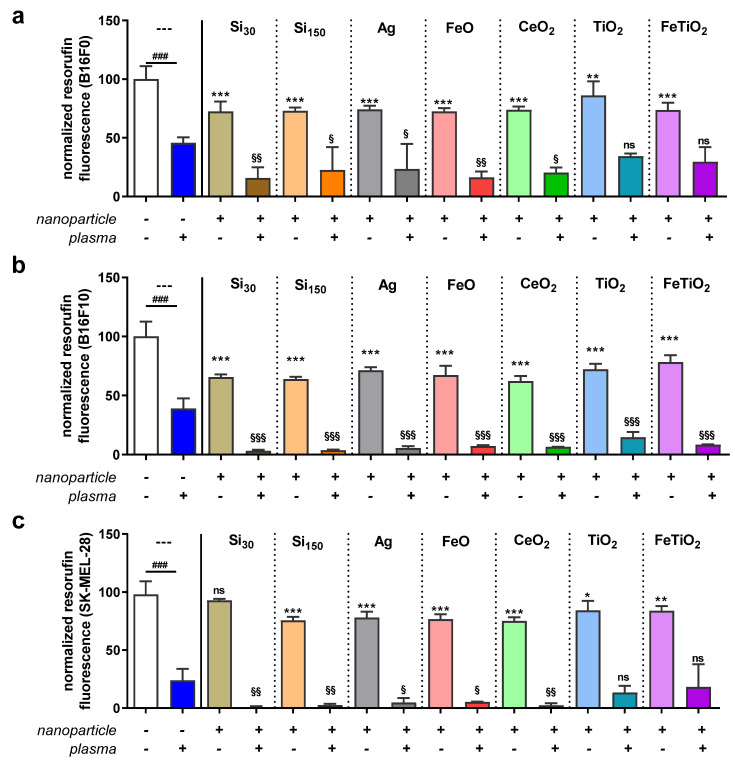
Combined cytotoxicity of nanoparticle exposure and gas plasma treatment. B16F0 (**a**), B16F10 (**b**), and SK-MEL-28 (**c**) cells were exposed to nanoparticles (10 µg/mL) immediately followed by gas plasma treatment. Data are the mean and standard error from three experiments comparing all nanoparticle samples without gas plasma treatment to vehicle control without gas plasma treatment (asterisks: *) and all nanoparticle samples with gas plasma treatment to vehicle control with gas plasma treatment (paragraph symbols: §) using one-way analysis of variance, and untreated vehicle controls versus gas plasma-treated vehicle control (hashtag: #) using the *t*-test.

**Figure 4:**
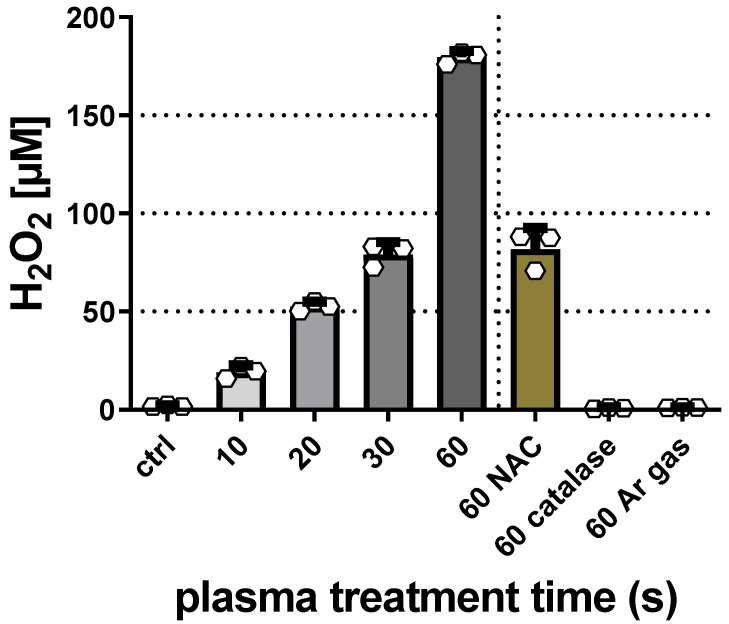
Reactive oxygen species (ROS) generation via gas plasma treatment. Phosphate-buffered saline (PBS) was exposed to gas plasma for 10 s, 20 s, 30 s, and 60 s, with the latter in the presence or absence of catalase or n-acetylcysteine (NAC), before quantification of hydrogen peroxide (H_2_O_2_). Untreated PBS and PBS exposed to argon gas alone (plasma: off) served as negative controls.

This study aimed to investigate the combined cytotoxicity of gas plasma treatment and nanoparticles in three melanoma cell lines in vitro. To this end, silica nanoparticles of two sizes (i.e., SI_30_ and Si_150_) and metal oxide nanoparticles that included silver, iron oxide (FeO), cerium oxide (CeO_2_), titanium oxide (TiO_2_), and 10% iron-doped titanium oxide (FeTiO_2_) were utilized (Table 1). After exposure to nanoparticles, the overall growth pattern of non-metastatic murine B16F0, metastatic murine B16F10, and metastatic human SK-MEL-28 (Figure 1) cells was reduced when investigated 16 h after particle addition. To next see whether nanoparticle toxicity followed a dose-dependent pattern, a serial dilution of particles was added to the B16F0 (Figure 2a), B16F10 (Figure 2b), and SK-MEL-28 (Figure 2c) cells, and the metabolic activity was investigated 24 h later, confirming a concentration-dependent cytotoxic effect in a pilot experiment. Subsequently, we tested the combined efficacy of gas plasma treatment with nanoparticle exposure, with the data differing to a minor extent from those obtained in the pilot experiments. In B16F0 cells, gas plasma treatment alone (20 s treatment time) reduced the metabolic activity by approximately 50% when investigated at 24 h, while exposure to the seven different types of nanoparticles (10 µg/mL) was lesser than that (Figure 3a). Upon combination of nanoparticle exposure immediately followed by gas plasma treatment, significantly increased combined cytotoxic effects were observed for Si_30_, Si_150_, Ag, FeO, and CeO_2_ but not TiO_2_ and FeTiO_2_ nanoparticle–gas plasma combination treatments. In B16F10 cells, the results were overall similar to those found in the B16F0 cells, with the exception of TiO_2_ and FeTiO_2_ nanoparticle–gas plasma combination treatments showing a significantly increased cytotoxicity when compared to gas plasma treatment alone (Figure 3b). In SK-MEL-28, the gas plasma treatment alone was more cytotoxic than in the murine cell lines, while the combination effects were similar to those found in the B16F0 cells (Figure 3c). Finally, a treatment time-dependent ROS generation of gas plasma was found (Figure 4).

## 4. Discussion

This study investigated the combined effect of gas plasma treatment and seven types of nanoparticles in three melanoma cell lines and found the combined toxicity of both treatment modalities. While combining nanoparticles and plasma is not necessarily novel by itself, the usage of several types and investigation of several cell types might be a valuable contribution to some researchers following such an approach.

Nanoparticle toxicity in melanoma cells has been described for several nanoparticle types. For example, silver nanoparticles were previously added to B16 melanoma cells, and dose-dependent cytotoxicity and intracellular ROS production were reported [27]. Along similar lines, silica nanoparticles were found to exert cytotoxic activity in murine melanoma cells as well [28]. Analogous findings were obtained for FeO nanoparticles [29] as well as CeO_2_ particles, with the latter having the unique feature of acting as both radio-protectors and radio-sensitizers when combined with radiotherapy [30]. TiO_2_, in turn, has been reported to be a ROS-promoting agent that, in combination with photo-activation, shows promising anticancer activity [31]. An anti-melanoma effect of Fe–TiO_2_ has not been reported so far and was more pronounced in B16F0 compared to B16F10 cells. Both cell types differ more than 100 fold in their capacity to form lung nodules in vivo, which ultimately correlates with overall survival in mice [32]. On the molecular level, the increased metastatic potential of B16F10 was attributed to the expression of the acidic actin βm, which results in enhanced invasiveness in collagen and cell motility in vitro, and ultimately also in vivo [33]. The B16F10 cells are also characterized by a weaker vinculin expression [34], which is turn is an adapter protein for, e.g., integrins to promote cell adhesion and non-invasiveness [35].

It is interesting to note that an ROS promotion or potentiation has been described with nanoparticle exposure in melanoma cells. Gas plasma systems are equally capable of generating delicate mixes of ROS/RNS that, in contrast to nanoparticles and pharmacological agents, target the tumor cells from the extracellular milieu [36]. Hence, several groups have tested the combined toxicity of nanoparticle and gas plasma before, albeit commonly only using a single type of nanoparticle [37,38]. For instance, gold nanoparticles were found to combine with gas plasma-mediated cytotoxicity in G361 melanoma cells [39], and similar results were obtained in glioblastoma cells [40] and human HCT-116 colorectal cancer cells [41] in vitro. Even more, gas plasma treatment was proposed to accelerate the uptake of gold nanoparticles into glioblastoma cells in two separate studies [42,43]. Investigating the combined toxicity of gold nanoparticles and gas plasma treatment in human U937 lymphoma cells, it was shown that the combined effect was a result of intracellular glutathione (GSH) depletion and modulation of oxidative stress [44]. As a mechanism of action, it has been noted many times that gas plasma treatment generates several types of short-lived ROS/RNS in the plasma gas phase, which subsequently enter the bulk liquid and finally—in the absence of target biomolecules—deteriorate to long-lived species [45]. The levels of H_2_O_2_ were measured in PBS that does not contain proteins, and hence the measurements overestimated the final H_2_O_2_ concentration compared to fully-supplemented cell culture medium by about 30% [25]. Nevertheless, at sufficient concentrations, the gas plasma-derived ROS/RNS promotes tumor cell inactivation [46], which was proposed to engage, e.g., mitochondrial stress [47,48,49] and target antioxidant defense mechanisms [50,51,52]. While a range of modes of action has been described for nanoparticle-mediated cytotoxicity, both mitochondrial alterations and ROS production are frequently observed phenomena [53,54,55], which may have contributed to the combined cytotoxicity found in this study. This combined toxicity was less pronounced in the TiO_2_ and FeTiO_2_ combination regimens, and the reasons for this remain the subject of future studies.

In this study, particular combined toxicity was found for iron, cerium oxide, silica, and silver nanoparticles. For iron nanoparticles, combined toxicity with gas plasma treatment was previously found in breast cancer cells in vitro [56]. Studies using both gas plasma exposure and cerium oxide nanoparticles have not been reported yet. A range of studies are available for silver nanoparticles, demonstrating combined cytotoxicity with gas plasma treatment in Hs 294T human melanoma cells [57] and even antimicrobial efficacy in bacterial phytopathogens [58]. Gas plasma exposure in glioblastoma cells also led to enhanced silver nanoparticle uptake rates and enhanced cytotoxicity [59]. The combined use of silica nanoparticles, especially with the two different sizes used here, with gas plasma for cancer treatment has not been reported so far.

While it was hypothesized to see combined toxicity with the types of particles used in combination with plasma treatment, other types, such as non-toxic gold nanoparticles, would have been interesting to investigate as well. Future studies would also benefit from intracellular ROS measurements and kinetic ROS monitoring during plasma or nanoparticle treatments to clarify ROS’s involvement to a more considerable extent. Moreover, combination treatments with a range of plasma treatment times and isobologram analysis might reveal additional effects that are lacking in the current study due to the single plasma treatment time used. Monitoring nanoparticle uptake might give additional insight into the toxicity mechanisms observed in this study. Subsequent studies are warranted to elaborate on the mechanistic action and in vivo relevance of these findings.

## 5. Conclusions

A combined cytotoxic effect of gas plasma treatment and five types of nanoparticles was found in three melanoma cell lines in vitro. In this setting, silica, silver, iron oxide, and cerium oxide particles gave the best responses across all cell lines.

## Figures and Tables

**Table 1:** Types and properties of nanoparticles investigated in this study.

Type of Nanoparticle	Hydrodynamic Diameter in Water (nm)	Zeta Potential
*Silica (Si) nanoparticles*		
Si_30_	33	−15
Si_150_	141	−21
*Metal oxide nanoparticles*		
Ag	12	−29
FeO	200	−20
CeO_2_	135	−49
TiO_2_	466	−36
FeTiO_2_	216	−44

## Data Availability

The data presented in this study are available on request from the corresponding author.
